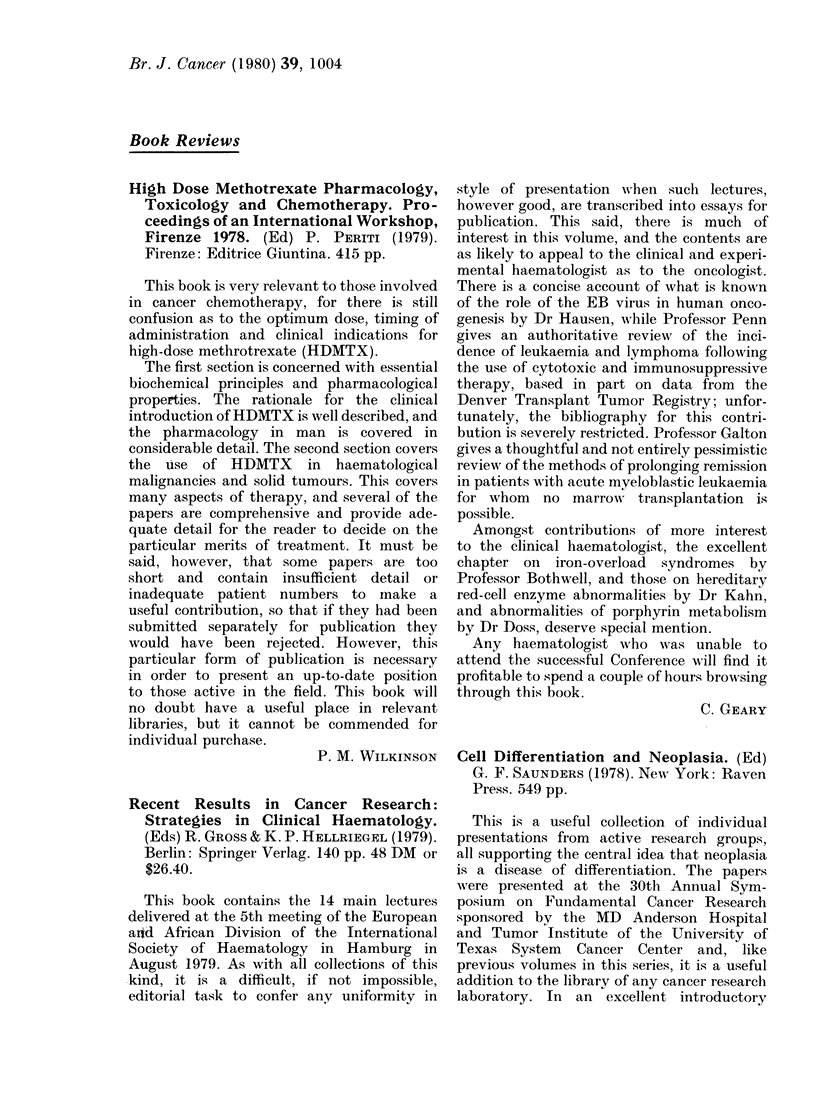# High Dose Methotrexate Pharmacology, Toxicology and Chemotherapy. Proceedings of an International Workshop, Firenze 1978

**Published:** 1980-06

**Authors:** P. M. Wilkinson


### *Book Reviews*

**High Dose Methotrexate Pharmacology, Toxicology and Chemotherapy. Proceedings of an International Workshop, Firenze 1978.** (Ed) P. Periti (1979). Firenze: Editrice Giuntina. 415 pp.

This book is very relevant to those involved in cancer chemotherapy, for there is still confusion as to the optimum dose, timing of administration and clinical indications for high-dose methrotrexate (HDMTX).

The first section is concerned with essential biochemical principles and pharmacological properties. The rationale for the clinical introduction of HDMTX is well described, and the pharmacology in man is covered in considerable detail. The second section covers the use of HDMTX in haematological malignancies and solid tumours. This covers many aspects of therapy, and several of the papers are comprehensive and provide adequate detail for the reader to decide on the particular merits of treatment. It must be said, however, that some papers are too short and contain insufficient detail or inadequate patient numbers to make a useful contribution, so that if they had been submitted separately for publication they would have been rejected. However, this particular form of publication is necessary in order to present an up-to-date position to those active in the field. This book will no doubt have a useful place in relevant libraries, but it cannot be commended for individual purchase.

P. M. Wilkinson